# Epistemological parameters in the recent era of nuclear organization and function

**DOI:** 10.1091/mbc.E26-04-0196

**Published:** 2026-07-23

**Authors:** Thoru Pederson

**Affiliations:** ^a^Department of Biochemistry and Molecular Biotechnology University of Massachusetts Chan Medical School, Worcester, MA 01605; Yale University

## Abstract

The sweeping progress in the nuclear organization-function field over the past two decades constitutes a major advance, to be sure. This work significantly impacts molecular, cell, and developmental biology and has uncovered numerous clinical implications. Here I offer perspectives around the epistemological axis in this field of ours, *viz.* what we knew and when, and what we know (or think we know) now.

## Abstract (executive summary)

- How both the DNA and other elements of the cell nucleus are spatially and functionally organized was pondered a century ago, with some prescient hints.
- Over the next 75 years, considerable progress was made and then, in the last two decades, a transformative leap occurred.
- This review describes this recent major “inflection point”, one that resulted from methodological breakthroughs and new ideas (“in that order” to honor a famous quote) and which now promise to take this field to the tissue and organ level, particularly the brain, and to capitalize on new findings of clinical potential now at hand.

## INTRODUCTION

From the time chromosomes were first discovered, in mitotic and meiotic cells (refer to Gall, 1996), how they are organized during interphase was a black box, to the extent it was even pondered. In some cases, the presentation of meiotic chromosomes was so dramatic, for example, the “lampbrush” chromosomes of amphibian oocytes whose investigation was pioneered by H.G. Callan, Oscar Miller, and Joseph Gall, that it seemed inconceivable that this elaborate organization could be accommodated within the confines of the interphase nucleus of typical somatic cells. Electron microscopy enabled visualization of the elementary chromatin fiber (Ris, 1961) but could not, of course, address the larger scale of chromosomal interactions. The same was true in cases where premature chromosome condensation was observed in interphase nuclei after fusion with mitotic cells (Rao and Johnson, 1970) or by exposure of cells to hypertonic medium (Robbins *et al.*, 1970).

And yet all through that era we knew that the location of a genetic locus within the nucleus could influence its expression, as had been most impactfully demonstrated earlier in the phenomenon of *Drosophila* position-effect variegation (Muller, 1930). That discovery could be rightly viewed as nothing less than the beginning of the entire nuclear organization-function field. Years later, investigation of many other breakage and recombination events added a new question, namely, in cases of reciprocal translocations, how do the ends find one another? The classic case here was, of course, the so-called Philadelphia chromosome (Nowell and Hungerford, 1960), later found to not be a single breakpoint as initially thought but a reciprocal translocation (Rowley, 1973). The fact that this resulted in a fusion protein that is oncogenic was heuristically fortunate in that it catalyzed the cytogenetic frontier of the cancer field. Much later, it was found that the probability of these reciprocal translocations relates to the initial proximity of the two loci (Roix *et al.*, 2003), not entirely unanticipated yet important to be established (Pederson, 2003).

The foregoing was the setting when the subsequent phase of our field that is mainly addressed here got underway. In this review, I summarize what I regard as major features in the evolution of the nuclear structure-function field beginning at about 2000. It is not intended to be a typical comprehensive review article but rather to emphasize trends in our thinking and understanding. My orientation will thus be an epistemological axis, as it has been in many of my other perspectives (e.g., Pederson, 2024a). I had a biochemistry professor who emphasized that we should not only know what is known but also how it came to be known, and I guess his point has stuck with me.

## THE BEGINNINGS

The modern era of the local spatial restriction of interphase chromosomes was pioneered by Thomas Cremer and collaborators who elegantly confirmed Boveri's initial observations of “chromosome territories” (Boveri, 1909; Cremer *et al.*, 1982a, 1982b). Another issue that soon arose was whether the interphase chromosomes were tethered in some way or could move about. An important study revealed that the chromosomes of yeast cells display diffusional movements, although within confinement volumes (Marshall *et al.*, 1997). The alternative concept of a “nuclear matrix” had arisen but did not take hold in most quarters (Pederson, 2000). Moreover, at this time many of us in the field recalled the so-called Rabl configuration, named after its first observer (Rabl, 1885), where in at least some cell types the interphase chromosomes are aligned with the telomeres at the nuclear envelope and the centromeres more oriented to the interior. Key subsequent advances included the demonstration of a radial configuration of active and inactive sites in many cell types (Croft *et al.*, 1999) and specific interactions of chromosomal regions with nuclear structures such as the lamina (Pickersgill *et al.*, 2006) and the nucleolus (Nemeth *et al.*, 2010; von Koningsbruggen *et al*., 2010). These milestones greatly advanced the breadth of our understanding of nuclear organization, and they were made possible by new tools- ones that continued to subsequently be expanded and diversified.

## MEIOSIS, A DISCOVERY LAUNCH PAD

The next major step was in the late 1990s in the laboratory of Nancy Kleckner at Harvard, who had already made major contributions to chromosome biology. How meiotic chromosomes pair had been investigated in the classical era, and key factors had been discovered later, but the details remained a quest for Kleckner and her lab. Her group came up with an ingenious method for capturing interchromosomal interactions in meiosis (Dekker *et al.*, 2002), and it immediately became apparent that it could, in principle, be applied to somatic cells. The first author of this seminal paper, Job Dekker, subsequently joined the faculty of my institution and undertook the refinement of “chromosome conformation capture” (abbreviated “3C”) and its application to genome biology overall (Mirny and Dekker, 2022). The impact was of considerable breadth as to the number of groups that adopted this platform and deployed it in many productive ways. In due course Dekker and collaborators created an array of refinements, the first two of which were branded “4C” and “5C.” Among these advances was the important introduction of the concept of “Topologically Associated Domains (TADs). Then “Hi-C” arrived, a particularly transformative advance led by Erez Lieberman-Aiden in collaboration with Dekker that combined the chromosome capture technology with bioinformatics and computational biology (Lieberman-Aiden *et al.*, 2009).

## THE US NATIONAL INSTITUTES OF HEALTH “COMMON FUND”

In 2013, I became aware of this special NIH program when contacted by two colleagues in our field. Administered by the NIH Office of the Director, this special fund seeks ideas from time to time for broad initiatives. Two of my colleagues at the NIH, Tom Misteli and Thomas Reid, informed me that they and others were advancing a proposal for support of a large-scale attack on how the spatial organization of the genome impacts gene expression, development, and human disease. I was invited to support their proposal and enthusiastically did so, as did others. After the various proposals had been reviewed by the NIH Director's advisers, the one for a “4D Nucleome” initiative was approved.

## MEETINGS- HERALDS OF A NEW FIELD

As publications in this evolving field appeared in increasing numbers, the typical parallel “signature of promise” occurred when a nascent field takes a forward step, *viz.* the advent of major meetings. These included the launch in 1998 of a Cold Spring Harbor Laboratory biennial meeting on Nuclear Organization and Function, then a similar EMBO biennial meeting starting in 2005 and held in alternating years with the former. A third, smaller meeting that nonetheless turned out to be important was one at the Jackson Laboratory, Bar Harbor, Maine, launched in 2001 and called the Genome Architecture Consortium, where biophysics and the nanoscale level were emphasized (O'Brien *et al.*, 2003). These three meetings drew together many groups that had not previously been connected. In addition, the nucleus became increasingly included in meetings that would have previously focused exclusively on the cytoplasm, for example, one on molecular machines that included due emphasis on nuclear ones (Bullock *et al.*, 2020).

Maintaining our epistemological theme, another key development was the NIH-funded ENCODE project, launched 12 years before the 4D Nucleome Initiative. Its ambitious goal of mapping the totality of genomic regulatory elements and their chromatin accessibility had been impressively realized by the time the 4D Nucleome launched and was enormously enabling for the latter. We shall return to this important connection between the two endeavors.

Although the genome itself was the focus in the 4D Nucleome Initiative getting underway in this period, there was also a growing awareness of the importance of various nuclear bodies that facilitate gene expression. Through the 1990s and 2000s, there had been considerable progress on these, particularly the nucleolus (Pederson, 2011), and as the 4D Nucleome Initiative got going, the nuclear bodies field continued to thrive and advance. This period of the nuclear bodies field not only benefitted from the 4D Nucleome but contributed to it in certain ways. And, as with the 4D Nucleome, the nuclear bodies field has developed its own community and biennial meeting (Kaufman *et al*., 2022; Chen *et al.*, 2024), with the next to be held this October.

## PHYSICS COMES TO THE NUCLEUS, AND VICE-VERSA

In two of his earliest papers, Francis Crick investigated the viscosity of the cytoplasm (Crick, 1950; Crick and Hughes, 1950). He was unlikely to have considered doing so for the nucleus, as no methods for that were at hand, though ones are today (e.g., Cheng *et al.*, 2025). But more than half a century later, in the period described in this article, the physical and biophysical theater of the nucleus had advanced in parallel with the molecular and genomic dimensions (Pederson, 2014; Pederson and Marko, 2014; Pederson *et al.*, 2015). Key studies revealed that the fluid viscosity of the nucleus is much lower than many had assumed (Wachsmuth *et al.*, 2000) and that RNA traffic in the nucleus is entirely diffusional (Politz *et al.*, 1998, 1999). Important advances were also made on how nuclear structure responds to mechanical stress (Miroshnikova and Wickstrom, 2022; Todorovski *et al.*, 2023; Vahabikashi *et al.*, 2023) as well as on factors that protect against this in certain cases (Alabi *et al.*, 2025). But most transformative as to physics, by now an epochal paradigm shift had occurred.

## CONDENSATES ARRIVE ON THE NUCLEAR SCENE

When I was a first-year graduate student, three molecular biologists had recently been appointed to the faculty, and they quickly populated the weekly seminar schedule with their like. I vividly recall the visits by the likes of Jean-Pierre Changeux, Arthur Pardee and Gordon Tomkins (they were really more biochemists than molecular biologists, but this was a period of great synergy between the two disciplines). Their seminars on the catalytic versus regulatory subunits of the enzymes they were studying. I paid no attention to how these stuck together, nor, frankly, did they—it was these enzymes’ feedback regulation that was on the wing. But, as we now know, heterotypic protein complexes can form in other ways, ones that were unanticipated back then.

In a study that will always be regarded as a milestone in our field, the amplified nucleoli in *Xenopus* oocytes were revealed to undergo a dynamic liquid-like phase transition (Brangwynne *et al.*, 2011). This phenomenon had been reported earlier for a type of cytoplasmic granules, and, both for the nucleus and cytoplasm, these findings set off a major “course correction.” To date, this physical attribute has been defined for the nucleolus, transcription sites, nuclear speckles, Cajal bodies and heterochromatin. Its basis was discovered to reside in low complexity domains of participating proteins.

## FISHING EXPEDITIONS

Many of us have had a review committee label a grant application as such, and there are surely kinder and more constructive ways. But a different kind of FISHing expedition occurred in 1969–1970, and it was anything but a pejorative term but rather, a revolutionary method in molecular cell biology (Gall and Pardue, 1969; Pardue and Gall, 1969; John *et al.*, 1969; Pardue *et al.*, 1970). In due course it underwent three advances. The first was the replacement of radioactive probes by fluorescent ones (Langer *et al.*, 1982; Matera and Ward, 1992), with F thus added to In Situ Hybridization to give FISH. The second was the advent of ever more facile chemical synthesis of oligos and the third was extraordinary innovation arising in the period covered here, one of which was “Oligopaint” (Beliveau *et al.*, 2015; Reboul *et al.*, 2025). These and related advances contributed mightily to the ability to image the 3D organization of chromosomal loci at enhanced scale.

## CRISPR- IN ITS OTHER DEPLOYMENT

The enormous breakthrough of CRISPR-based gene editing can never be overstated. But a group at UCSF had the idea that it could also be turned to the localization and dynamics of specific chromosomal loci in live cells (Chen *et al.*, 2013). This had been achieved previously in pioneering advances by Andrew Belmont and colleagues using GFP-tagged *lac* operator arrays (Robinett *et al.*, 1996), and later by others who deployed fluorescent TALE's (Ma *et al.*, 2013; Miyanari, Ziegler-Birling and Torres-Padilla, 2013; Thanisch *et al.*, 2013). But the CRISPR-based approach (Chen *et al.*, 2013) had advantages in terms of its ease. My group and collaborators subsequently expanded CRISPR-based tracking of genomic loci to achieve greater spectral range (Ma *et al.*, 2015, 2016a) and sensitivity (Ma *et al.*, 2018), as well as to reveal new insights into the live cell kinetics of CRISPR action (Ma *et al.*, 2016b) and interphase chromosome dynamics (Ma *et al.*, 2019).

## TODAY AND BEYOND

Now I turn to some of the most recent developments, offering thoughts on new dimensions and directions in our field that seem particularly promising and/or provocative to me.

## A NEW ERA OF SYNERGY: THE ENCODE-4D NUCLEOME NEXUS

The completion of the decade-long 4D Nucleome Initiative in August of 2025 as an NIH Common Fund program has not meant that these investigators have turned their attention elsewhere- few, if any, have. There is, for example, significant momentum on neurobiological aspects as well as an increasing disease focus-both of which were getting underway in the 4D Nucleome Initiative by 2024. As just one of many examples, the human congenital malformation known as pulmonary agenesis has been associated with a complex set of genomic rearrangements including the de novo formation of a TAD at a particular genomic locus (Melo *et al.*, 2021). Additional directions emerging from the 4D Nucleome Initiative can also be sensed in the recent summary of its decade of many accomplishments (Dekker *et al.*, 2025). These advances synergize with the recently reported 2.5-fold expansion of the human *cis*-regulatory elements identified previously in ENCODE, as well as a three-fold expansion of the murine ones (Moore *et al.*, 2026). Moreover, this all comes at a time when we increasingly understand how transcriptional enhancers’ cooperativity is based on their propinquity in the folded genome (Friman and Bickmore, 2026; Hansen *et al.*, 2026) and are raising ideas about enhancers sometimes looping to ones nearby, instead of to promoters- the latter having been accepted as the “standard model” (Struhl, 2025).

## Mapping the nuclear proteomic landscape

The initial proteomics analyses of the nucleolus (Andersen *et al.*, 2002) and interchromatin granule clusters (Mintz *et al.*, 1999; Saitoh *et al.*, 2004) were important first steps and were followed during the period covered here by proximity labeling methods to capture proteins within 10–20 nm of a given nuclear body or specific genomic site (Kaewsapsak *et al.*, 2017; Chen *et al.,* 2018; Gao *et al.*, 2018; Fazi *et al.*, 2019; Dopie *et al.*, 2020; Liu *et al.*, 2024; Tsue *et al.*, 2024). Another version of these methods, developed by Wei Zhang and colleagues at Synthcell, Taipei, Taiwan, and termed “Microscoop,” involves training a light beam on a very small, desired site within the nucleus and thus spatially limiting the reporter's activation (refer to Chen *et al.*, 2024). Other key advances include ways to restrict or eliminate rogue free radical behavior, previously looming from the photochemistry of these methods. And the recent extraordinary revolution in cryo-electron tomography has also found application to the nucleus (Kechagia and Medalia, 2026). All these emerging methods are ushering in a bold new frontier of subnuclear proteomics at the nanoscopic scale.

## CONDENSATES, REDUX

Adding up the nucleolus, certain transcription sites, almost all the nuclear bodies, and heterochromatin, it emerges that a substantial part of the intranuclear mass behaves in this way. It is also possible that we do not yet completely understand the full dimension of condensate physical chemistry (in the nucleus or anywhere in the cell). There are recent findings that suggest this might be the case (e.g., Shinn *et al.*, 2026), as do recent studies of unanticipated capillary effects at the edges of these bodies, which change the equations of state (Gouveia *et al.*, 2022). Integrating these new aspects into the nuclear condensates field constitutes a new frontier, beyond the initial liquid-liquid phase separation views- as heuristically catalytic as they were.

## PUSHING THE GENOME AROUND

I have been surprised that Hi-C has not been more intensively applied to ask how transgenes adjust the 3D-nucleome. Small insertions might not be expected to appreciably alter the Hi-C map, for example moving a locus across a TAD boundary, and it is perfectly possible that even with larger insertions (i.e., transgenic experiments with cells or mice), deleterious effects may have passed unnoticed. But as clinical applications of AAV-based gene therapy advance, this issue becomes pressing. This technology often involves non-integrating vectors, but not always. Indeed, in the latter cases, transcriptionally active sites are selectively targeted (Russell, 2003). I don't think anyone knows why and, after all, we do know that transcriptionally inactive sites are by no means buried. As always, when a puzzle like this arises, it is a place on which to drill down.

## HYPERMUTATION, GENOME STABILITY AND MEMORY

Like the other aspects of our field listed above, here is one that again has seen tremendous progress. Nonetheless, there remain cases of promising potential. For example, somatic hypermutation, long known as the basis of B-lymphocyte-mediated immunity, has recently been connected to intranuclear chromatin organization (Schoeberl *et al.*, 2025), and this beckons further pursuit. Another recent finding is that DNA double-strand breaks leave a heritable genomic mark at the site of their repair (Bantele *et al.*, 2025). This is as intriguing a recent finding as any in our field and, like the somatic hypermutation finding, has obvious clinical potential. A third area ripe for investigation is a longstanding mystery in reproductive biology, namely that mammalian oocytes display paradoxically low mutation rates. There is increasing evidence that this may relate to unique features of oocyte genome organization (Dudko *et al*., 2025). A fourth area of great importance is around the fourth “D” in the term “4D Nucleome”, viz. how this organization is stably inherited in dividing cell populations (when it is, and why when it is not) and locked in non-mitotic ones. The latter seem to get “special treatment.” What is it? Histone marks have been implicated (Paldi and Cavalli, 2026; Paldi *et al.*, 2026), but is there something more going on?

## LET'S NOT FORGET THE LAMINA

Not to push Paul Simon's song “Mother and Child Reunion” too far as metaphor, but I think of the telophase-reforming lamina and the subsequently organizing G1 genome in this way, the latter finding its “remembered footing,” as it were. The heritability of genome-lamina contacts is on the one hand known, and on the other a mystery as to how it works. I think this is as important a frontier as any in our field. In what safe is the early G1operating manual, to soon be deployed, kept for those 30–45 min. of mitosis? Could a clue lie in how the cytoplasm senses the disassembly and reassembly of the nucleus? After all, we know that the nuclear lamina and the cytoplasmic intermediate filament system are not exactly “unaware” of one another (e.g., Vahabikashi *et al.*, 2022).

## IN CLOSING

In these latter sections I have, of course, only touched on the many new avenues unfolding in our field, and readers will have their own favorites. And let's face it, there are likely to be new developments that come out of the blue. I recall from high school Latin that the word science comes from the verb “to know” (*scire*), but years later it dawned on me that our word “science” descends from the Latin verb's present participle, *sciens* = knowing. And so, just because we have proudly learned so much over the past decade in our field and possess some insight into how we got to know it, we must humbly admit that we most certainly don't know everything. That is our ticket for the future, in both meanings of “admission.”

## Abbreviations used:

AAV: adenovirus-associated virus
ENCODE: encyclopedia of DNA elements.
