# Supplementary material for: Epistemological parameters in the recent era of nuclear organization and function
Source: Mol Biol Cell. 2026 Jul 23;37(9):re9. doi: 10.1091/mbc.E26-04-0196 (PMC13525607; doi:10.1091/mbc.E26-04-0196)
Supplement: Supplementary file 1 [file reviewer_reportE26-04-0196.pdf]

Epistemological parameters in the recent era of nuclear organization and function

Thoru Pederson

Corresponding author(s): Thoru Pederson, University of Massachusetts Chan Medical School

**Review Timeline:**	Submission Date:	2026-05-04
	Editorial Decision:	2026-05-28
	Revision Received:	2026-06-09
	Accepted:	2026-06-10

Editor-in-Chief: John Aitchison

Transaction Report:

(Note: With the exception of the correction of typographical or spelling errors that could be a source of ambiguity, letters and reports are not edited. The original formatting of letters and referee reports may not be reflected in this compilation.)

RE: Manuscript #E26-04-0196
TITLE: "Epistemological parameters in the recent era of nuclear organization and function"

Dear Dr. Pederson:

Congratulations on this excellent work, and thank you in advance for your thorough and thoughtful responses to the reviewers. I am pleased to inform you that your manuscript has been provisionally accepted for publication in Molecular Biology of the Cell (MBoC) pending completion of the minor revisions listed below.

To finalize acceptance, please address the following items:

Thank you for the contribution and the thoughtful article. The reviewers were very positive and suggested a few minor clarifications for you to consider in revising the work for a broader audience. I would appreciate that you consider reviewer 1's points 3 and 4 and in particular reviewer 2's point 3.

Once these revisions are submitted and verified, we will proceed with formal acceptance.

Thank you again for your contribution to MBoC.

Sincerely,
Patrick Lusk
Monitoring Editor
Molecular Biology of the Cell

Dear Dr. Pederson,

The review of your manuscript, referenced above, is now complete. The Monitoring Editor has decided that your manuscript requires minor revisions before it can be published in Molecular Biology of the Cell, as described in the Monitoring Editor's decision letter above and the reviewer comments (if any) below.

A reminder: Please do not contact the Monitoring Editor directly regarding your manuscript. If you have any questions regarding the review process or the decision, please contact the MBoC Editorial Office (mboc@ascb.org).

When submitting your revision include an authors' response letter that details, point-by-point, how the Monitoring Editor's and reviewers' comments have been addressed. (The file type for this letter must be "response letter"; do not include your response to the Monitoring Editor and reviewers in a "cover letter.") Please bear in mind that your response letter will be published with your paper if it is accepted, unless you have opted out of publishing the review history.

Authors are allowed 180 days to submit a revision. If this time period is inadequate, please contact us immediately at mboc@ascb.org.

In preparing your revised manuscript, please follow the instructions in the Information for Authors (www.molbiolcell.org/info-for-authors). In particular, to prepare for the possible acceptance of your revised manuscript, submit final, publication-quality figures with your revision as described.

To submit the response letter, revised version, and figures, please use this link (please enable cookies, or cut and paste URL):
Link Not Available

MBoC offers the option to publish your work with immediate open access. Open access can increase the discoverability and usability of your research. If you would like to publish your paper with immediate open access but did not select this option during initial submission, please contact us at mbc@ascb.org.

Authors of Articles and Brief Communications whose manuscripts have returned for minor revision ("provisional acceptance") are encouraged to create a short video abstract to accompany their article when it is published. These video abstracts, known as Science Sketches, are up to 2 minutes long and will be published on YouTube and then embedded in the article abstract. Science Sketch Editors on the MBoC Editorial Board will guide you as you prepare your video. Information about how to prepare and submit a video abstract is available at www.molbiolcell.org/science-sketches. Please contact mboc@ascb.org if you are interested in creating a Science Sketch.

Thank you for submitting your manuscript to MBoC. Please do not hesitate to contact this office if you have any questions.

Sign up for MBoC's new content alerts here https://tinyurl.com/3bcsyk3e

Sincerely,

Eric Baker
Journal Production Manager
MBoC Editorial Office
mbc@ascb.org

Reviewer #1 (Remarks to the Author):

The author presents a review covering the conceptual and technical landmarks that have paved the way to our understanding of nuclear organization, with special emphasis on chromatin architecture and the achievements of the 4D Nucleome initiative.

I found the manuscript interesting, easy to follow and a valuable guide to, as the author´s former professor declared, learn "what is known but also how it came to be known" about the organization of the nucleus.
Nevertheless, I would like to make a couple of comments and suggestions to the author:

1- Page #5: I think it would be interesting for a broader audience to include the acronym "3C" when mentioning the chromosome conformation capture method, as it is a very commonly used term to refer to the technique, and it might help understand why later methods were also called 4C, 5C, Hi-C, etc.

2- I would suggest for the titles of the sections to be a bit more informative/descriptive if possible, as some of them come across as a bit cryptic. Adding a small sentence after some of them summarizing what the topic is about would be helpful for the reader.

3- Page #10: I am missing references to the relevant opto-proteomic papers, please add them. I would also like to suggest including what in my opinion is one of the most exciting and promising avenues of research: the in situ characterization of the nuclear and chromatin organization by the combination of cryo-ET, AI-assisted template matching and integrative methods, what has been called by some "Visual proteomics".

4- In the "Today and beyond" section, I am also missing a mention to the study of mechanical force transduction to the nucleus - potentially affecting chromatin architecture, epigenetic landscape and expression - as a modern and promising avenue of research.

5- It is usually customary to somehow apologize to the authors of relevant studies that (understandably) due to space constraints could not be cited in the review, so I would suggest to include a paragraph to that effect in the Acknowledgements section.

Reviewer #2 (Remarks to the Author):

This is an enjoyable and easy read from a prominent figure in the field of nuclear/genome organization. It is part review and part commentary that also has autobiographical elements. It looks back on key findings in the field and introduces future areas of consideration, at least with respect to the interests of the author. The article is written in a "musing" style that is in general scholarly but is deliberate in not digging too deeply on any subject. I don't have any major criticisms of the work. I do, however, have a couple of points for the author to consider in making the manuscript more informative and accessible to a broader audience.

Point 1: The article seems directed to individuals in the field who are expected to have a basic understanding of the fundamentals. For example, terms like "TADs" and "enhancers" are dropped into the text without any context or definition. This is fine but I think this article should be available to the broadest possible audience and adding a few key definitions or at least pointing the reader to useful reviews may serve such a purpose.

Point 2: The author deals with subjects that span many decades and there is a broad past versus present organization, however, there is also a lot of bouncing around across these decades. I'm wondering whether the author would find it valuable to include a figure of a timeline where some of the key advances (in their opinion) were made and by whom to provide a resource for the reader.

Point 3: The section on the 4D nucleome contains a paragraph that speculates that Francis Collins may have had an inherent

bias for funding the 4D nucleome proposal. Although I'm certain this was not the intention, I don't think it is appropriate to speculate on Francis Collins' decision making and this paragraph should either be removed or altered.

Point 4: There is a discussion of the advent of "chromosome conformation capture" but it isn't clear whether the author is attributing this to Nancy Kleckner or Job Dekker (or both). Please clarify. A suggestion would be to also use this paragraph to be more specific about the "truly transformative advancement of the field" - at a minimum, this could be the place to introduce TADs.

Other points in random order:
1) "as Sydney Brenner had stated" - what is the quote here?
2) "As one example, a recent study...can be penetrating". A couple more words about what was shown in this study would be nice.
3) Some places where I felt a reference was missing: "these methods involve training a light beam..."; "Ways to restrict or eliminate rogue free radical behavior"; "to date, this physical attribute has been defined..."; there is a discussion of capillary effects of condensates but I think that recent work suggesting unique pH environments in the nucleolus from Pappu could also fit well in this paragraph;
4) Please check references 83-85 in text for accuracy.

RE: Manuscript #E26-04-0196R
TITLE: "Epistemological parameters in the recent era of nuclear organization and function"

Dear Dr. Pederson:

I am pleased to accept your manuscript for publication in Molecular Biology of the Cell.

Sincerely,
Patrick Lusk
Monitoring Editor
Molecular Biology of the Cell

Dear Dr. Pederson:

Congratulations on the acceptance of your manuscript! Thank you for publishing your work in Molecular Biology of the Cell (MBoC).

Within 10 days, an unedited PDF of your manuscript will be published on MBoC in Press, an early-release journal version. The date your manuscript appears on this site is the official publication date.

Your copyedited and typeset manuscript will be scheduled for publication in the next available issue of MBoC. Once your paper is ready for review, our production team will notify you. In the summer of 2024, our production provider will introduce an online proofing solution, which will streamline the process of checking and validating author changes. This will result in improved typesetting quality, enhanced digital content, and a faster overall process.

We look forward to publishing your paper in MBoC.

Sincerely,

Eric Baker
Journal Production Manager
MBoC Editorial Office
mbc@ascb.org
